# Open-source modular field-programmable gate array system for two-photon mesoscope enabling multiarea, multidepth neural activity recording and lifetime imaging

**DOI:** 10.1117/1.NPh.13.1.015013

**Published:** 2026-03-03

**Authors:** Riichiro Hira, Fumiya Imamura, Hiroto Imamura, Yuki Yoneyama, Takehisa Handa, Osamu Fujioka, Che-Hang Yu, Satoshi Suitoh, Reiko Hira, Atsushi Kamoshida, Shigeki Kato, Kazuto Kobayashi, Hiroki Shiwaku, Hidehiko Takahashi, Spencer LaVere Smith, Akihiro Funamizu, Yoshikazu Isomura

**Affiliations:** a Institute of Science Tokyo, Graduate School of Medical and Dental Sciences, Department of Physiology and Cell Biology, Tokyo, Japan; b High Performance Artificial Intelligent System Research Team, Center for Computational Science, RIKEN, Saitama, Japan; c University of Tokyo, Institute for Quantitative Biosciences, Laboratory of Neural Computation, Tokyo, Japan; d University of Tokyo, Graduate School of Arts and Sciences, Department of Life Sciences, Tokyo, Japan; e Institute of Science Tokyo, Department of Psychiatry and Behavioral Sciences, Tokyo, Japan; f TRIONIX Inc., Saitama, Japan; g University of California Santa Barbara, Department of Electrical and Computer Engineering, Santa Barbara, California, United States; h KYOCERA SOC Corporation, Kanagawa, Japan; i Graphical Design Lab, Saitama, Japan; j Fukushima Medical University, Institute of Biomedical Sciences, Department of Molecular Genetics, Fukushima, Japan

**Keywords:** FPGA, four-plane imaging, two-photon, multiplexing, mesoscope, lifetime imaging, open-source, cerebral cortex

## Abstract

**Significance:**

Large field-of-view (FOV) two-photon microscopy enables simultaneous recording across multiple brain regions, but larger FOVs lengthen raster scans and limit temporal resolution. A modular, open-source solution that increases imaging speed and adds fluorescence-lifetime capability on standard systems would broaden access to mesoscale neural measurements.

**Aim:**

We aimed to develop and validate an open-source, modular field-programmable gate array (FPGA)-based acquisition platform and a circular delay-path (CDP) module that together enable multiarea, multidepth mesoscale two-photon imaging and large-FOV two-photon fluorescence lifetime imaging (2p-FLIM) using an 80 MHz laser.

**Approach:**

We built an FPGA system that digitizes photomultiplier signals at 3.2  GS/s and integrated it with a CDP module for a Diesel2p mesoscope. The CDP temporally multiplexes excitation for four focal planes; the FPGA demultiplexes and reconstructs images. Lifetime imaging was implemented on the same platform.

**Results:**

The system enabled simultaneous recording of >10,000 neurons across the bilateral dorsal cortex at up to four depths and demonstrated large-FOV 2p-FLIM. All hardware and software are open-source and compatible with existing two-photon microscopes.

**Conclusions:**

This modular, open-source FPGA + CDP system increases throughput of large-FOV two-photon imaging and adds lifetime contrast without specialized lasers, facilitating multiscale *in vivo* studies and broad biomedical applications.

## 1 Introduction

Large field-of-view (FOV) two-photon microscopy has advanced systems neuroscience by enabling simultaneous recording of thousands of neurons across multiple brain regions.^1^^–^^5^ Two-photon microscopy requires the laser to be focused on a single point in space and time; therefore, a wider FOV leads to longer scan durations. Imaging speed can be improved either by faster point scanning or by multifocal imaging. Acousto-optic deflectors (AODs),^6^^,^^7^ tomographic methods,^8^ and temporal focusing^9^^–^^12^ can also increase the sampling rate but remain challenging to implement over large FOVs. Multifocal excitation using a beam splitter,^13^ a spatial light modulator (SLM),^14^^,^^15^ or a spinning-disk^16^^,^^17^ can raise the frame rate. A delay-line strategy combined with high-speed sampling can further accelerate acquisition by several-fold and is compatible with large FOV two-photon imaging.^3^^,^^5^^,^^18^^,^^19^ Extensions of this approach have achieved even faster scanning.^12^^,^^20^^–^^22^ Delay line strategies usually rely on low-repetition-rate lasers because the longer interval between pulses allows distinct temporal delays to be inserted for efficient multiplexing. Introducing such delays is a key route to faster imaging in large FOV two-photon microscopes. However, high-speed sampling in this context requires detailed knowledge of the detection electronics, and high-power, low-repetition lasers remain uncommon. An add-on module that uses the standard 80 MHz laser in existing systems to create a multifocal source would therefore markedly increase throughput and accelerate progress in systems neuroscience.

In addition to the fluorescence intensity, a two-photon microscope can measure fluorescence lifetimes by comparing the arrival time of fluorescence photons with that of the excitation pulses.^23^^–^^25^ There are two main approaches. Time-correlated single-photon counting (TCSPC) provides the highest temporal resolution^26^^–^^28^ and digitizes the photomultiplier-tube (PMT) signal at high sampling rates to reconstruct decay curves digitally.^29^^–^^34^ TCSPC excels when photon counts are low, whereas high-speed digitization is more scan-efficient for bright specimens that produce many photons, although it requires rapid digital processing. Open-source software that can handle these high-throughput data streams would bring significant benefits not only to neuroscience but also to biology and medicine.

In this study, we developed a laser multiplexing mechanism and an open-source software module for high-speed sampling and then integrated them with the Diesel2p mesoscope.^3^^,^^4^^,^^35^ The module samples the PMT signal at 3.2  GS/s without dead time, and at each 80 MHz laser pulse, the field-programmable gate array (FPGA) computes fluorescence values from four independent focal points in parallel. With this setup, we imaged neural activity in a mouse brain at four focal planes simultaneously and also demonstrated large field-of-view two-photon lifetime imaging using the same system. Because the fast analog sampling device is modular and can be added to any two-photon microscope, it promises broad benefits across biomedical research.

## 2 Materials and Methods

### 2.1 Animal Preparation

All experiments were approved by the Institute of Science Tokyo (A2021-290C6, A2024-060C2) and were carried out in accordance with the Fundamental Guidelines for Proper Conduct of Animal Experiment and Related Activities in Academic Research Institutions (Ministry of Education, Culture, Sports, Science and Technology, Japan). We used adult male and female double-transgenic mice (AI162 or TIT2L-GC6s-ICL-tTA2^36^ × Slc17a7-IRES2-Cre^37^) and wild-type mice. These mice were kept under an inverted 12  h/12  h light–dark schedule (lights off at 12:00 AM; lights on at 12:00 PM) in their home cages to match experimental timing.

### 2.2 Neonatal AAV Injection

Neonatal viral injection was done on postnatal day (P)1-2.^38^ The pups were anesthetized by hypothermia on an ice bed. A quartz glass pipette (outer diameter 50  μm) was inserted into the cortex to a depth of 250 to 300  μm below the pial surface. A total of 4  μL of AAV–DJ–Syn–jGCaMP8s^39^ was delivered over 1 min.

### 2.3 Surgery

Craniotomy over the dorsal cerebral cortex was performed under isoflurane anesthesia (1.5% to 1.8%). Body temperature was maintained using air-activated heat packs, and corneas were protected with ophthalmic ointment. The scalp overlying the dorsal cortex was removed, and a custom head-fixation imaging chamber was mounted to the skull with a self-curing dental adhesive (Super-Bond, L-Type Radiopaque; Sun Medical) and luting cement (Fuji Lute). The surface of the exposed skull was subsequently coated with clear acrylic dental resin (Super-Bond, Polymer Clear; Sun Medical) to prevent drying. Mice were then returned to their home cages after recovery.

### 2.4 Cranial Window Implant

Three days after head-plate surgery, we performed a large cranial window implant. The glass plug used to cover the dorsal cortex was made with a 6  mm×6  mm, #3 coverslip and a 7  mm×7  mm, #0 coverslip with two corners cut (Matsunami Glass, Kishiwada, Japan). The two coverslips were bonded using optical adhesive (Norland Optical Adhesive 63; Norland Products, Cranbury, New Jersey, United States). After anesthesia, 20% mannitol (5  μL per g body weight, intraperitoneal) was administered to reduce intracranial pressure and atropine sulfate (0.216  μg per g, intraperitoneal) to reduce saliva and mucus secretion. The head plate was secured in a stereotaxic frame. Previously applied acrylic resin was removed using a dental drill, followed by marking of the bregma and stereotaxic coordinates. The skull along the outline of the cranial window as well as the coronal and sagittal sutures was thinned with the drill. The frontal and parietal bones were lifted in four separate pieces. The glass plug was then placed on the exposed cortex, enabling optical access to the entire dorsal cortex, and sealed to the bone with cyanoacrylate glue and dental resin cement. Finally, the mouse was administered mannitol (10  μL per g, intraperitoneal) and meloxicam (0.38  μL per g, subcutaneous) before returning to the cage to recover for 5 days.

### 2.5 Two-Photon Imaging

All two-photon imaging was conducted with a custom-made mesoscope, Diesel2p.^3^ For excitation, we used a fixed-wavelength fiber laser, ALCOR 920-2 (Spark Lasers, Martillac, France). The group-delay dispersion (GDD) compensation, determined by maximizing the fluorescence intensity of a sample ($\approx 60,000\;{\mathrm{fs}}^{2}$), was adjusted within the laser. A 2× or 3× beam expander (GBE02-B, GBE03-B, Thorlabs, Newton, New Jersey, United States) expanded the laser to fill the back aperture of the objective lens. Two independent scan engines (path 1 and path 2), each comprising two galvanometer mirrors (R6220H, Cambridge Technology; GS7X-AG, Thorlabs) and an 8 kHz resonant scanner (CRS8kHz, Cambridge Technology, Bedford, Massachusetts, United States), together with an electrically tunable lens (ETL, EL-12-30-TC-VIS-16D; Optotune, Dietikon, Switzerland) and a shutter (SHB05T, Thorlabs), were controlled with a PCI-6722 (National Instruments, Austin, Texas, United States). The scanner power supplies were custom-made. The PMT (H10770PA-40, Hamamatsu, Hamamatsu, Japan) signal was amplified with either an amplifier (cutoff frequency 200 MHz, gain 100; Edmund Optics #59-179, Barrington, New Jersey, United States) or a C5594 preamplifier (cutoff frequency 1.5 GHz, gain 36 dB; Hamamatsu Photonics, Hamamatsu, Japan). When using the C5594, the signal was passed through a BNC high-pass filter (EF515; Thorlabs) and attenuated with a 10 dB BNC attenuator (Tyclon, BA-PJ-10, Tokyo, Japan) to fit the digitizer’s dynamic range. The signal was digitized with ATS-460 (200  MS/s; AlazarTech, Quebec, Canada) or PXIe-5774 (3.2  GS/s; National Instruments). We used the PXIe-5774 board for time-multiplexing and lifetime imaging, and the AlazarTech board in our standard configuration without multiplexing. The synchronization signals from the resonant scanner and the galvo scanner were recorded at 200  MS/s. The PXIe-5774 digitizer was installed in a PXIe-1082 chassis, and the signals were transferred to the host PC via a PXIe-8301 Thunderbolt interface. The entire system was controlled by custom software written in LabVIEW 2019 (National Instruments).

Calibration of the imaging scale was carried out empirically with a fluorescent scale. The scale was made by laminating a fluorescent reference slide (FSK5; Thorlabs) to a resolution test target (R1L3S1P; Thorlabs) and fixing the assembly in a slide holder (MAX3SLH; Thorlabs). A goniometric stage with a rotation center height of 125 mm (Misumi, B58-60TLC, Chiyoda-ku, Japan) and a rotation stage (XXR1/M, Thorlabs) under the objective lens were used to adjust the fluorescent scale until it was parallel to the imaging plane, after which calibration images were acquired.

### 2.6 Excitation Point Spread Function (PSF) Measurements

The measurement and analysis procedures were described in detail previously.^3^ To evaluate the excitation point-spread function (PSF), sub-micrometer fluorescent beads (0.2  μm-diameter, Invitrogen F-8811, Carlsbad, California, United States) were embedded in a ∼1.2  mm-thick 0.75% agarose gel, and the gel was sealed with a cover glass (Matsunami, No. 1, 0.12 to 0.17 mm). Z-stacks ranging from 30 to 100  μm were acquired. The stage was moved axially in 0.2  μm increments (Thorlabs, KVS30). At each focal plane, 10 frames were acquired and averaged to yield a high signal-to-noise image. Due to the difference between the refractive index of the objective immersion medium (air) and the specimen medium (water), the actual focal position within the specimen shifted by Δfocus=1.35×Δstage.

### 2.7 Multiplexing

**Fig. 1:**
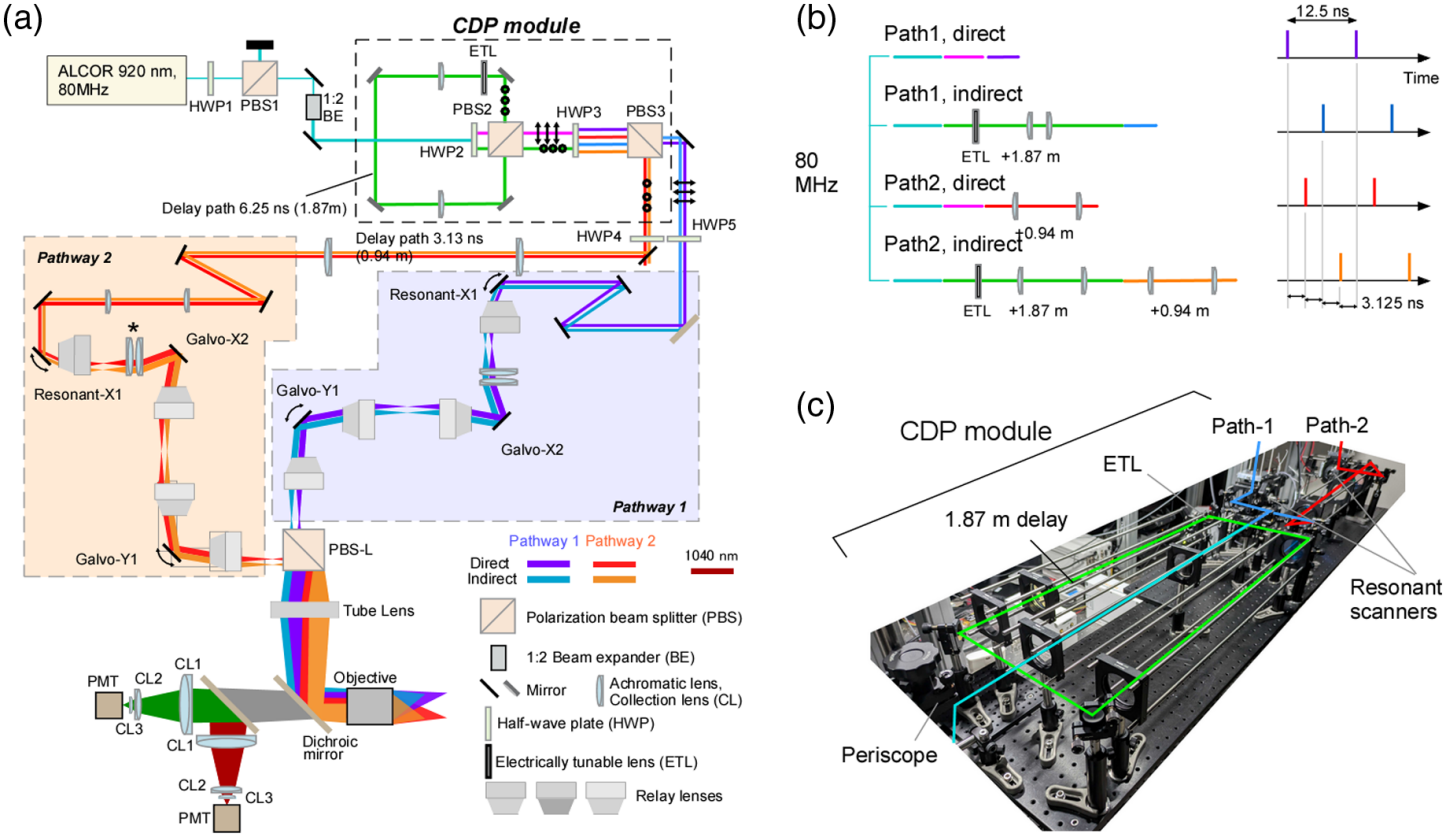
Diesel2p equipped with a CDP module that enables multiplexed imaging along four optical paths. (a) Schematic diagram of the Diesel2p optical layout with the circular delay-path (CDP) module inserted. (b) Conceptual drawing of the four paths represented as straight lines, showing spatial scale on the left and temporal scale on the right; each delay is relayed by a dedicated 4f system, and a 3.125 ns separation between paths 1 and 2 plus a 6.25 ns delay introduced by the CDP module yields an overall separation of 3.125 ns between consecutive paths. (c) A photograph of the CDP module.

The pulsed laser for excitation operated at 80 MHz (interpulse interval 12.5 ns). In a circular delay-path (CDP) module, an s-polarized laser was reflected by a PBS and routed through an additional 1.88 m loop (c×6.25  ns≈1.88  m). This produced a second pulse delayed by 6.25 ns. The CDP module contained an ETL to adjust focal depth. After recombination, the beam passed through a half-wave plate (HWP) and was split again by a second PBS. The beam propagated along either path 1 or path 2, with path 2 being 0.94 m longer, effectively dividing each source pulse into four pulses equally spaced by 3.125 ns (effective repetition rate 320 MHz). The two lateral paths enabled imaging at two different locations simultaneously. Furthermore, the presence of the ETL in the CDP module and achromatic lenses [asterisk in Fig. 1(a)] in path 2 made it possible to scan four planes at different areas and depths. The emitted fluorescence passed through the objective and a dichroic mirror and was detected by a PMT. The signals were then amplified and demultiplexed by an FPGA-based high-speed sampling module. Detailed instructions are provided in the Supplementary Material.

Power distribution among the four fields was adjusted as follows. Two sets of HWPs and polarizing beam splitters (PBSs) define two independent ratios: the ratio between the direct and indirect beams and the ratio between the two direct beams ($\text{path}\;1_{\text{direct}}$ and $\text{path}\;2_{\text{direct}}$). The ratio between the delayed beams ($\text{path}\;1_{\text{indirect}}$ and $\text{path}\;2_{\text{indirect}}$) is then fixed by the latter. Let the direct-to-indirect ratio be 1:x and the ratio of the two direct beams be 1:y. The indirect beams are therefore in the inverse ratio y:1. For an incident power M, the powers are

$$\text{path}\;1_{\text{direct}}=\frac{M}{\left(1+x)(1+y\right)},$$

$$\text{path}\;2_{\text{direct}}=\frac{My}{\left(1+x)(1+y\right)},$$

$$\text{path}\;1_{\text{indirect}}=\frac{Mxy}{\left(1+x)(1+y\right)},$$

$$\text{path}\;2_{\text{indirect}}=\frac{Mx}{\left(1+x)(1+y\right)},$$

giving the ratio

$$\text{path}\;1_{\text{direct}}:\;\text{path}\;1_{\text{indirect}}:\;\text{path}\;2_{\text{direct}}:\;\text{path}2_{\text{indirect}}=1:\;xy:\;y:\;x.$$

(Here D = direct, I = indirect.)

### 2.8 Simulation of Optical Pathways

For each of the four branched optical paths, we optimized the system in Zemax OpticStudio (v16) to ensure that the Strehl ratio exceeded 0.9 at both the field center and at a point 2.5 mm off-axis. The first and second direct paths were configured to allow a coherent laser beam to be incident on the resonant scanner, as in conventional scanning. By contrast, the first and second indirect paths were directed into the resonant scanner via a loop circuit. An electrically tunable lens (ETL EL-12-30-TC, Optotune) was incorporated into the loop circuit, thereby enabling the indirect paths to be scanned simultaneously in the axial direction. Because the ETL is configured with an offset lens such that its focal length becomes infinite at 0 mA, the focal positions of path 1 and path 2 are nearly identical when the ETL current is set to 0 mA. Starting from a 4f optical layout, we optimized the parameters by varying the distance between lenses to maintain a Strehl ratio above 0.8 even when the ETL current was altered. During optimization, the light source was set at 920 nm, and the beam diameter at the scanner was fixed at 3.6 mm. Although this diameter underfilled the resonant scanner’s 4 mm aperture, it was chosen to ensure sufficiently high laser transmission. The effective numerical aperture was determined via ray tracing. For each of the four optical-path configurations, the focal depth was determined by optimizing the spot radius while varying the distance from the glass window to the focus.

### 2.9 FPGA-Based Software

We configured five LabVIEW libraries to control data acquisition, storage, reconstruction, and export of imaging data. The “Diesel2p Acquisition” library provides APIs to control the PXIe-5774 FlexRIO digitizer (with on-board FPGA) and to acquire PMT waveforms. The “TDMS Async Write-Analog” library streams analog data to an SSD at high speed, and the “TDMS Async Write-H-sync” library saves H-sync timing data. The “Diesel2p File Read” library reconstructs and displays images from the stored analog and H-sync streams, and the “Diesel2p File Conversion” library batch-converts reconstructed images to TIFF files.

Using these libraries, we built four top-level VIs. “tc-All Data Path for Display Data.vi” renders images online via the Diesel2p Acquisition API. “All Data Path for Logging.vi” saves analog and digital data in TDMS format using the acquisition API together with both TDMS Async Write libraries. “Data Viewer.vi” plays back stored data as video via the File Read API. “File Conversion for Diesel2p.vi” exports images from stored TDMS data as TIFF. Each API is encapsulated as a subVI so that components can be reused across projects. Detailed VI and subVI structures are described in the Supplementary Material, and parameter descriptions are available through each subVI’s “Context Help.”

### 2.10 Real-Time Data Storage and Image Reconstruction

Data were saved in technical data management streaming (TDMS) format, a binary container designed for high-throughput streaming in LabVIEW. We stored FPGA-integrated fluorescence counts, the frame-start (V-sync) signal, and resonant-scanner sync (H-sync) signals in TDMS files. Offline reconstruction software reads these TDMS files, selects the scanner sync and channel (1 to 4), and writes per-path images to binary files. The binary data are then sine-wave corrected to compensate for the position-dependent scan speed of the resonant scanner and exported as 16-bit TIFF images. These images can be processed with MATLAB, ImageJ, or Python for registration and region-of-interest (ROI) detection, as in conventional two-photon workflows.

### 2.11 Image Processing

Image preprocessing was done using ImageJ (National Institutes of Health) and MATLAB (MathWorks, Natick, Massachusetts, United States) software. The bidirectional phase offset of the image was calculated, and sinusoidal distortions caused by the resonant scanner were corrected. The Turboreg plugin (Thevenaz et al., 1988^40^) on ImageJ was used for image registration. The registered images were analyzed using Suite2p^41^ to determine the location of the regions of interest (ROIs). The intensity of each ROI was calculated by subtracting the mean values of background pixels within 60  μm of the ROI, excluding the other ROIs from the mean value of the ROI. For each ROI, the 8 percentile pixel values within 15 s of the time window were obtained, which were then smoothed using a Gaussian filter with a standard deviation of 30 s. This was considered the baseline of neural activity and was subtracted from the original data to set the baseline to 0.

### 2.12 Single-Photon Signal Measurement

Full-speed data were recorded at 3.2  GS/s in the on-board memory of a PXIe-5774 digitizer, allowing individual photon signals to be resolved. Because the memory capacity is limited, each acquisition lasted ≈200  ms. Each PMT trace was processed by applying a threshold and aligning the waveform at the downward crossing point, which we interpret as a single-photon or multi-photon event. Urea crystals were used as the sample. Apart from the sample and laser power, all electronic settings were identical to those used in the *in vivo* and fluorescence-lifetime imaging experiments.

Although all components in the signal path were matched to 50 Ω, the small oscillatory component (“ringing”) was found probably because of minor impedance mismatches or high-frequency resonances in the analog detection chain [Fig. 3(b)]. In principle, additional analog damping elements could be introduced to suppress this ringing, but such measures would compromise the high-frequency signal content. Because multiplexed lifetime imaging functioned robustly with the IRF containing this residual ringing, we decided to keep the current configuration.

### 2.13 Impulse Response Function and Fluorescence Lifetime Imaging

We used Convallaria (standard FLIM specimen). The 80 MHz excitation pulses are separated by 12.5 ns, which the high-speed digitizer divides into 40 temporal bins (Δt=0.3125  ns). Because the digitizer can store data from only four windows at a time, we acquired 10 sequential images with different window positions and concatenated them to create a 40-frame lifetime stack with a temporal resolution of 0.3125 ns per frame. For each pixel, this procedure yielded a vector, $\left[y=y_{0},y_{1},\ldots ,y_{39}\right]$. To obtain the impulse-response function (IRF), we recorded the second-harmonic signal from a urea-crystal slide, giving, $\left[u=u_{0},u_{1},\ldots ,u_{39}\right]$. For convolution, we formed a periodic extension of the IRF, $\overset{¯}{u}=[u\;u\;u]$, to avoid wrap-around at the 12.5 ns boundary.

The fluorescence decay was modeled as the convolution of the IRF with a mono-exponential plus offset, sampled at Δt:

y^(A,B,τ)=u˜*f,with  f(t)=Ae−t/τ+B,  t≥0.

We estimated AAA, BBB, and τ by least-squares:

minA,B,τ>0 ∑k=039(yk−y^k(A,B,τ)).

Only pixels brighter than a preset threshold were fitted, and the fitted τ values were rendered as the fluorescence-lifetime image.

Because photon detection is a counting process, the noise on $y_{k}$ is more accurately described by Poisson statistics rather than additive Gaussian noise. To account for this, we additionally modeled the photon-counting process with a Poisson noise model. For a given pixel, the measured counts in bin k were assumed to be independent Poisson random variables with mean, i.e., the mean photon count in each bin is given by the IRF–convolved mono-exponential decay plus offset as defined above. In this formulation, the exponential term $e^{-t/\tau }$ specifies the probability density for the photon arrival time following an excitation pulse, and convolution with the IRF yields the expected detection time distribution. The Poisson model then governs how many photons are observed in each temporal bin when this stochastic arrival process is repeated over many excitation cycles.

The log-likelihood of observing a histogram $\left[y=y_{0},y_{1},\ldots ,y_{39}\right]$ for a given set of parameters (A,B,τ) is

$$\log\;L(A,B,\tau |y)\overset{39}{\underset{k=0}{\sum }}[yk\;\log\;\lambda_{k}(A,B,\tau )-\lambda_{k}(A,B,\tau )-\log(yk!)].$$

As the term $\underset{k}{\sum }\;\log(yk!)$ does not depend on the parameters, we estimated A, B, and τ by minimizing the negative log-likelihood

$$\arg\;\underset{A,B,\tau }{\min}\;\overset{39}{\underset{k=0}{\sum }}[\lambda_{k}(A,B,\tau )-yk\;\log\;\lambda_{k}(A,B,\tau )].$$

Only pixels brighter than a preset threshold were fitted, and the resulting maximum-likelihood estimates τ were rendered as the Poisson-noise-corrected fluorescence-lifetime image.

### 2.14 Phasor analysis

Phasor analysis was performed as follows. The impulse response function (IRF, 40 bins spanning 12.5 ns) was first normalized to the zero–one range. With the laser repetition period T=12.5  ns, the angular frequency was defined as ω=2π/T, and the phase of each bin k from 0 to 39 was set to $\theta_{k}=2\pi k/40$. The Fourier coefficients of the IRF were then obtained as

$$g_{0}=\frac{\underset{k}{\sum }y_{\text{norm}}(k)\cos\;\theta_{k}}{\underset{k}{\sum }y_{\text{norm}}(k)},\;s_{0}=\frac{\underset{k}{\sum }y_{\text{norm}}(k)\sin\;\theta_{k}}{\underset{k}{\sum }y_{\text{norm}}(k)}.$$

The fluorescence decay trace of every image pixel was likewise normalized to the zero–one range, and its coefficients were calculated as

$$g_{1}=\frac{\underset{k}{\sum }I_{\text{norm}}(k)\cos\;\theta_{k}}{\underset{k}{\sum }I_{\text{norm}}(k)},\;s_{1}=\frac{\underset{k}{\sum }I_{\text{norm}}(k)\sin\;\theta_{k}}{\underset{k}{\sum }I_{\text{norm}}(k)}.$$

Instrument response was corrected by treating the IRF coefficient $C_{0}=g_{0}+is_{0}$ and the pixel coefficient $C_{1}=g_{1}+is_{1}$ as complex numbers and computing $C=C_{1}/C_{0}$, which gives g and s after separation into real and imaginary parts. The phase φ was obtained from arctan (s/g), and the lifetime was calculated as

$$\tau =\frac{\tan\;\varphi }{\omega }\times 10^{9}\;(\mathrm{ns}).$$

All calculations were executed automatically with a custom MATLAB R2024a script.

### 2.15 Optimization of Detection Windows by Genetic Algorithm

A genetic algorithm was used to choose four temporal accumulation windows from each forty-bin decay waveform (total length twelve point five nanoseconds) so as to maximize the accuracy of single-exponential lifetime estimation with the four-gate method. Before optimization, each specimen and IRF waveform was circularly shifted by 19 bins to place the reference bin at bin 0 (t=0), a constant baseline was subtracted, and the result was stored as a 40-sample vector with 0.3125-ns spacing. Each gene consisted of eight ordered bin numbers to $b_{1}$ to $b_{8}$, interpreted as four windows $\left(b_{1},b_{2}\right)$, $\left(b_{3},b_{4}\right)$, $\left(b_{5},b_{6}\right)$, and $\left(b_{7},b_{8}\right)$. The time $t_{g}$ for a window was taken as the average of its two bin centers multiplied by 0.3125 ns. The observed intensities $Y_{g}$ for the specimen and R(τ) for the IRF were defined as the mean intensity in the corresponding bins. Lifetime τ for a given gene was obtained by fitting the model

$$Y_{g}=\int_{t_{g}}^{t_{g}+\Delta t}[R(\tau )*\exp(-t/\tau )]dt,$$

and finding the value of τ that minimized the residual sum of squares

rss(τ)=∑g=14(Yg−Y^g(τ))2,

Within the interval 0.3125 ns, using a one-dimensional search (*fminbnd*) with a tolerance of $1\times 10^{-4}$. The ground-truth lifetime τ_truth for fitness evaluation was the 40-bin single-pixel full fit obtained with the conventional method, and fitness for each gene was defined as the Pearson correlation coefficient between τ and τ_truth across 120 randomly chosen pixels. The initial population contained 100 genes, and the population evolved for 100 generations. In each generation, the 20 genes with the highest correlation were retained unchanged as elites, and the remaining 80 were produced by selecting an elite at random and shifting one of its bin indices by ±1. Duplicate genes were removed to maintain diversity. The four-window configuration that achieved the highest correlation in the final generation was adopted for subsequent experiments. All computations were carried out automatically with custom MATLAB R2024a scripts.

### 2.16 Correction of Fluorescence Leakages

To estimate fluorescence leakage between channels, we imaged a brain slice expressing GCaMP with multiplexing disabled (single 80 MHz beam) and sampled the fluorescence every 0.3125 ns (40 bins per 12.5 ns period). Because the system records four channels in parallel, we acquired 100 frames per setting (four consecutive bins per acquisition), averaged them, and then repeated this procedure while stepping the acquisition window across the 40 bins. This yielded 40 mean images with pixel intensities that reflect the delay of the GCaMP6s signal relative to each laser pulse, given the combined response of the PMT, pre-amplifier, and A/D converter used under the same settings as in the *in vivo* and fluorescence-lifetime experiments. These temporal profiles were then employed to quantify leakage when four fields of view were imaged concurrently. For each laser pulse, the raw pixel values in the four channels were written as J=[J1,J2,J3,J4]⊤, and the true values in the absence of leakage as I=[I1,I2,I3,I4]⊤. We assumed a linear mixing model so that J=WI where W is a 4×4 constant matrix with off-diagonal elements that represent cross-talk coefficients determined from the time-resolved calibration data

$$w=\mathrm{circ}(1,a,b,c)=[\begin{array}{cccc} 1 & a & b & c\\ c & 1 & a & b\\ b & c & 1 & a\\ a & b & c & 1 \end{array}],$$

where a, b, and c are the leakage coefficients for signals that arrive one, two, and three steps earlier, respectively. Applying $I=W^{-1}J$ with this explicit inverse removes the leakage among the four channels while preserving the original signal amplitudes. After restoring the pixel values for each laser pulse, we reconstructed the images using the start trigger of each resonant scanner sweep. Because our system employs two independent resonant scanners, pixels acquired along the two optical paths do not correspond one-to-one in the raw data. Consequently, instead of correcting the leakage on the reconstructed images, we applied the channel-mixing correction on a pulse-by-pulse basis and then rebuilt the images from the corrected pulse data.

### 2.17 Data Analysis

We used Rastermap^42^ to visualize the simultaneously recorded neuronal activity. The parameters were set as follows: n_cluster=50, n_PCs = 64, time lag window = 20, and locality = 0.7. All other parameters were kept at their default values. Rastermap was executed by calling Python 3.1 from MATLAB. K-means clustering was performed using the built-in MATLAB function “*kmeans*” with K set to 8. We also tested values of K ranging from 4 to 10 and obtained qualitatively similar results. PCA was performed using the built-in MATLAB function “*pca*”.

### 2.18 Statistics

Wilcoxon’s rank sum test was used for pairwise comparison. Data were expressed as means ± S.E.M unless otherwise noted.

## 3 Results

### 3.1 Laser-Efficient Four-Way Beam Splitting

We designed optical pathways in which the 80 MHz laser with an inter-pulse interval of 12.5 ns is divided into four parts, with one pulse reaching the specimen every 3.125 ns. First, a loop optical path (1.88 m per loop) was constructed so that the s-polarized laser reflected by the PBS makes one loop. The ETL was placed in the loop for focal-depth adjustment under the objective. Two convex lenses (f=500) were added in the loop in a 4f configuration. A half-wave plate (HWP) was placed in front of the loop optical path to change the power ratio between the beam entering the loop and the beam transmitted straight ahead. The loop output and the straight-through beam were recombined with the PBS, passed through another HWP, and then split by a second PBS into two lateral imaging paths. The reflected s-polarized laser went to path 2, and the passing p-polarized laser went to path 1 [Fig. 1(a)]. In path 2, an additional 0.94 m delay optical path was introduced, and two convex lenses were added in this pathway in a 4f configuration. As a result, the four pulses passed through different optical paths with additional delays of 0, 0.94, 1.88, and 2.82 m of additional delay, creating focal points under the objective lens that were delayed by 3.125 ns [Fig. 1(b)]. The first and third pulses via path 1, and the second and fourth pulses via path 2, scanned separate lateral regions. Within each lateral region, the ETL introduced a focal-depth difference between the two pulses; this depth offset was adjustable. The image sizes of all pathways were the same because ETL was placed at a plane conjugate to the objective pupil. Furthermore, the laser power could be optimized in the two depths and in the two areas by rotating HWPs. This simple loop-based design therefore enabled efficient splitting of an 80 MHz laser into four interleaved beams for multidepth, multi-area imaging. We call this module a CDP module [Fig. 1(c)].

### 3.2 PSF Analysis for Four Pathways

We first characterized the beam shape in each of the four optical paths by measuring the two-photon point spread function with 0.2  μm beads, whereas the ETL was kept at its default setting. For the first direct, first indirect, second direct, and second indirect, we acquired point spread functions (PSFs) at three field positions: the field center, the right edge located 2.5 mm from the center (X-edge), and the upper right corner located 2.8 mm from the center (diagonal-edge). We confirmed that all four paths showed almost identical resolution. Resolution along the z-axis was slightly decreased away from the center, but the axial full width at half maximum (FWHM) remained below 10  μm. These observations confirm that each field retains the designed optical performance.

### 3.3 ETL Controls Focal Depth of Indirect Pathways

**Fig. 2:**
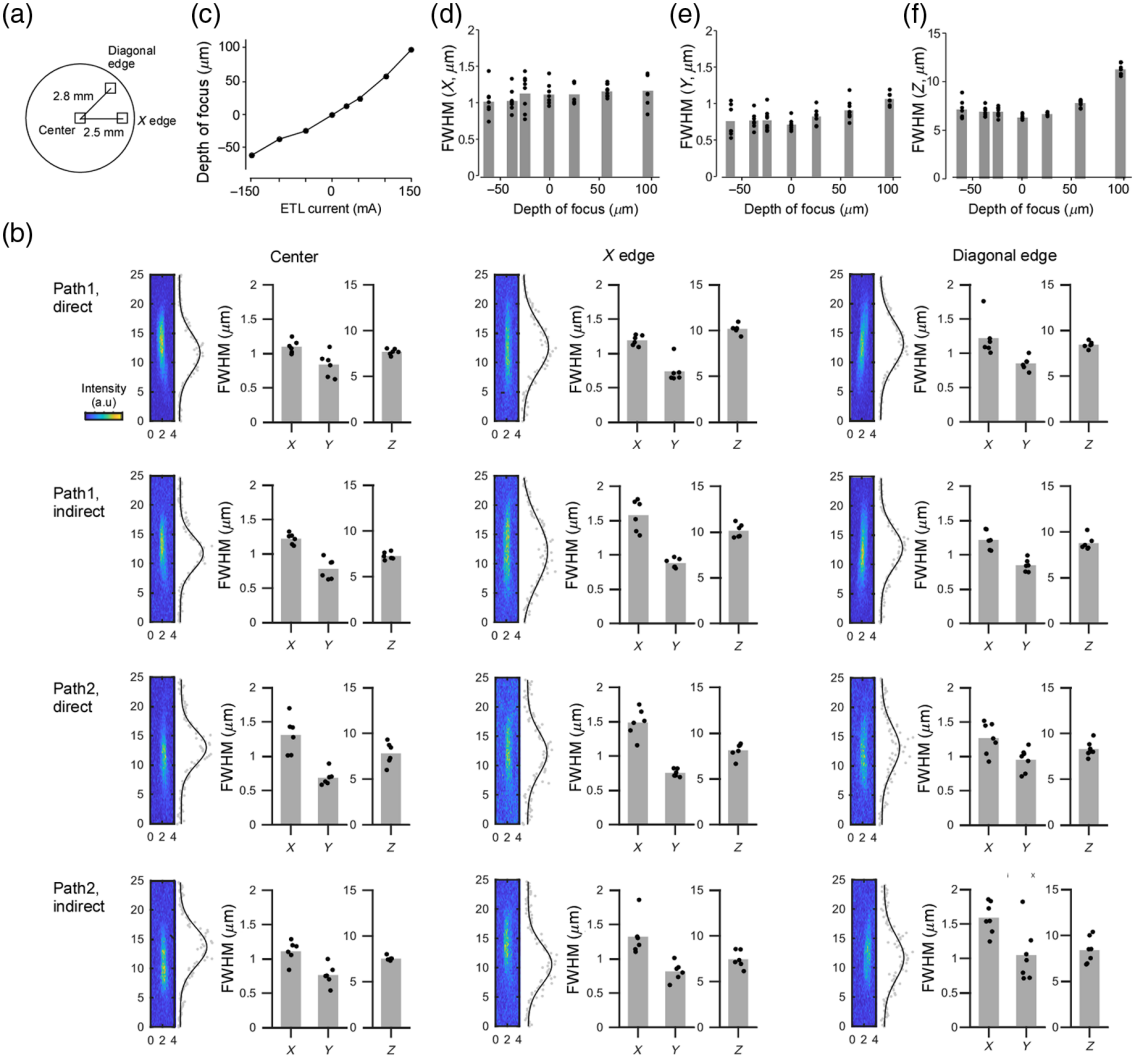
PSF analysis. (a) Point-spread functions (PSFs) were measured at three locations—image center, X-edge, and diagonal edge—for each of the four optical paths. (b) The full widths at half maximum (FWHMs) of the PSF in the X, Y, and Z directions are summarized for all four paths using 0.2  μm fluorescent beads; the left sub-panel shows a representative PSF with its axial intensity profile and Gaussian fit, whereas the right sub-panel lists the X-, Y-, and Z-axis FWHM values (all values in μm). (c) For path 1D, the focal depth was plotted against the driving current of the electrically tunable lens (ETL). (d)–(f). The horizontal (X,Y) and axial (Z) PSF FWHM as a function of focal depth. The sample was at the center position of the field of view.

Because the first and second indirect paths contain an ETL, their focal depths can be adjusted. We drove each lens from −150 to +150  mA and compared the focal depth and PSF full width at half maximum (FWHM). In path 1I (Fig. 2), the focal depth shifted by ≈0.54  μm per mA. Throughout this range, the lateral FWHM (X and Y) remained virtually unchanged, whereas the axial resolution broadened slightly. Overall, these results show that the ETL provides at least 164  μm of axial translation while preserving effective two-photon excitation efficiency.

**Fig. 3:**
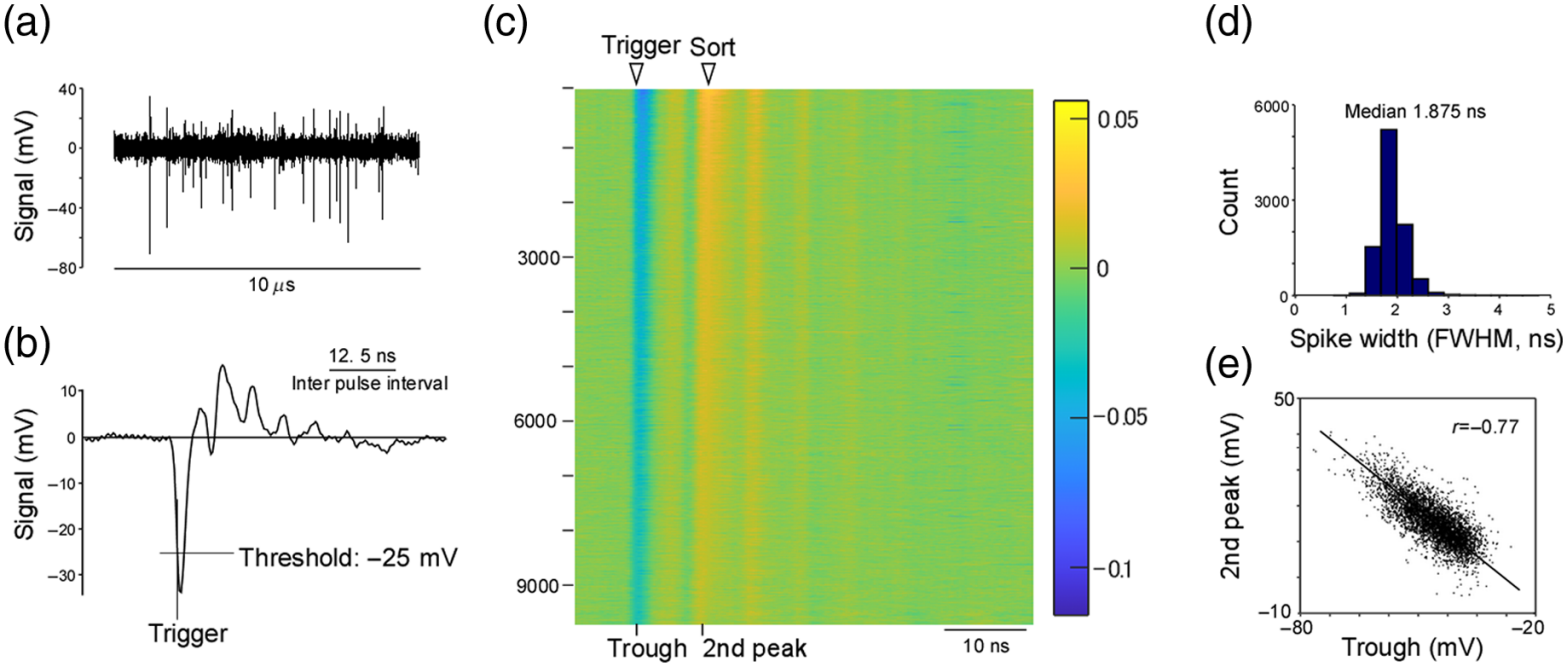
Single photon signal. (a) Analog PMT signal sampled at 3.2  GS/s over 10  μs; the urea crystal was excited at the minimum power that still produced detectable fluorescence, so each negative pulse is taken to represent a single photon. (b) Average waveform of the negative pulses in panel (a), showing a damped oscillation that follows the initial trough. (c). Color map of ≈10,000 aligned pulses used for the average in panel (b), sorted by the amplitude of the second positive peak; nearly every pulse exhibits the same temporal oscillation. (d) Distribution of the full width at half maximum of the negative pulses; the median is 1.875 ns. (e) Scatter plot of the time bins containing the first trough and the second positive peak for each pulse, revealing a strong correlation (r=0.77).

### 3.4 Separating Four Fluorescence Signals Using FPGA

To separate the four fluorescence signals on the FPGA, we exploited the fixed 3.125 ns delay between successive excitation pulses, which produces an identical 3.125 ns offset in the arrival time of the corresponding fluorescence at the PMT. Before implementing this demultiplexing, we verified that individual photon signals were fully contained within that window by imaging the second-harmonic generation of a urea crystal at very low laser power. The PMT output, amplified and filtered identically to *in vivo* measurements, was digitized at 3.2  GS/s; most laser pulses produced no response, and a few yielded isolated negative spikes followed by oscillations [Figs. 3(a)–3(c)]. The negative peak had a full width at half maximum of 1.8 ns [Fig. 3(d)], safely shorter than 3.125 ns, and the timing of the first through and second positive peak was highly correlated across pulses [r=0.77; Fig. 3(e)], indicating that the oscillation originated in the detection electronics rather than in photon statistics. Because this oscillatory tail was much smaller than the initial spike, it could be rejected by integrating within a suitably short window. Accordingly, the FPGA assigned four nonoverlapping 3.125 ns integration windows to the photomultiplier output, summed the photon counts for each pulse in real time, and streamed the results as four independent channels corresponding to path 1D, path 1I, path 2D, and path 2I. This pulse-by-pulse demultiplexing removed temporal cross-talk before image reconstruction and allowed simultaneous acquisition of four focal planes without sacrificing temporal resolution.

### 3.5 FPGA System

**Fig. 4:**
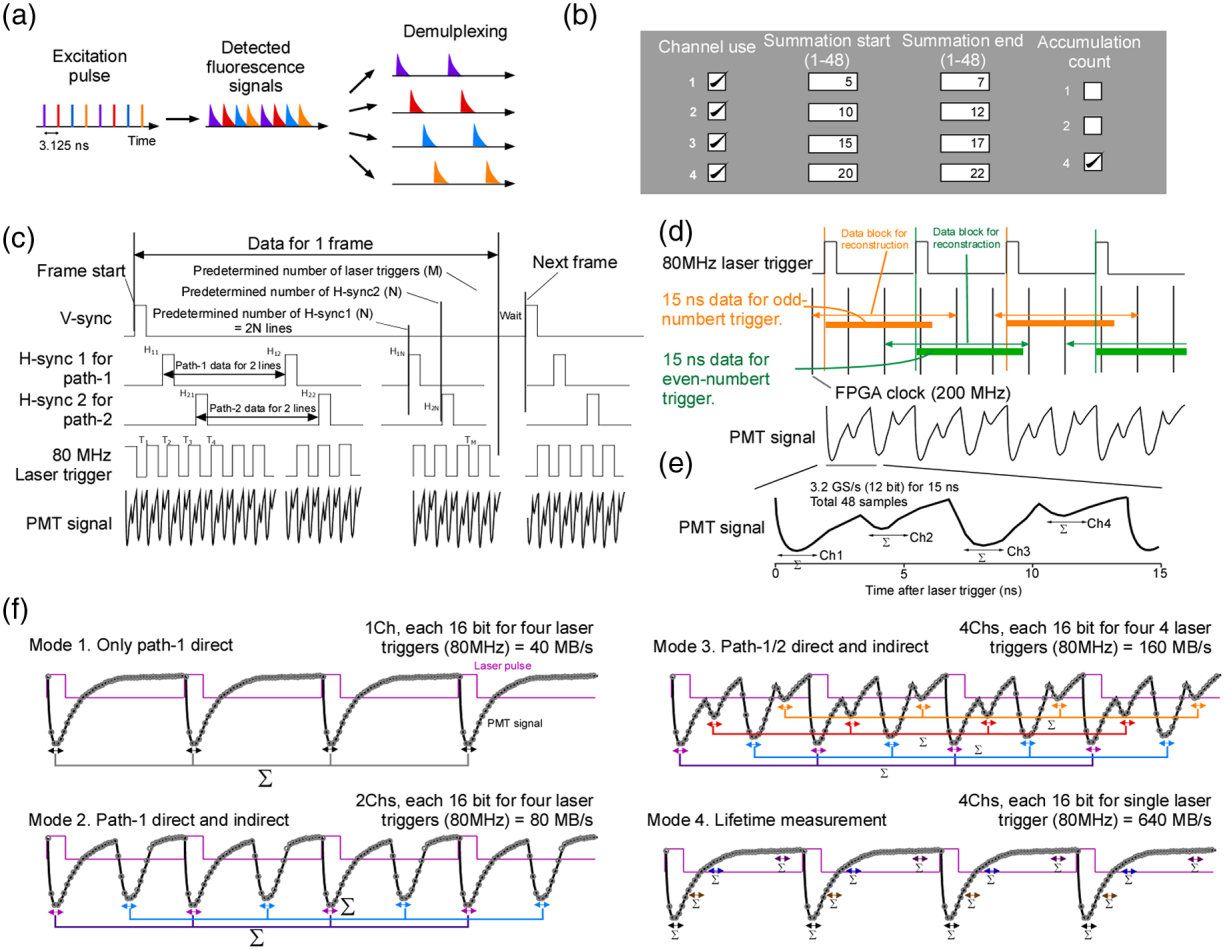
Operating modes of the FPGA digitizer and the underlying mechanism. (a) The system separates four fluorescence signals that arrive 3.125 ns apart. (b) The FPGA digitizer can operate in several different configurations. The user selects which of the four optical paths to use with checkboxes. For each channel, the user can specify which time window to integrate out of 48 time points (a total of 15 ns) measured from the laser pulse trigger. The user can also choose how many consecutive laser pulses to sum before saving the data. The available options are 1, 2, and 4 pulses. In the figure shown here, all four channels are enabled, and the digitizer outputs the summed signal from time points 5 to 7, 10 to 12, 15 to 17, and 20 to 22 for four consecutive laser pulses. As a result, the FPGA outputs four data streams to the host PC at 20 MHz. For more details, see the Supplementary Material. (c) A frame is defined by five inputs—vertical sync, two horizontal sync signals, the laser clock, and the PMT output. After a vertical-sync pulse marks frame start, the module acquires PMT data for the prescribed number of horizontal-sync events (N) and laser pulses (M). (d) Signal processing for odd and even laser pulses is interleaved (orange for odd, green for even). Forty-eight samples are stored per pulse, covering 15 ns so that fluorescence lifetimes extending beyond the 12.5 ns pulse period are fully captured. (e) Representative PMT waveforms recorded for the four optical paths. (f) Operating modes: mode 1 records a single path and, by summing four pulses, yields 20  MS/s; mode 2 records the direct and delayed branches of path 1; mode 3 records all four paths simultaneously; mode 4 records four-gate lifetime data.

The purpose of our system is to distinguish in the time domain the four fluorescence signals that follow four excitation pulses spaced 3.125 ns apart [Fig. 4(a)]. Some experiments do not need all four optical paths and often do not require single-pulse resolution, so the module can operate in several modes that compress the data stream and lighten later analysis [Fig. 4(b)]. Users set three parameters: the number of active channels, which temporal bins each path should sum, and the accumulation count that determines how many consecutive pulses are averaged for compression.

To verify single-photon signals, we first recorded at 3.2  GS/s, but at that rate, the two-gigabyte on-board memory fills almost immediately. To allow continuous recording, the FPGA integrates the photomultiplier output within four windows for every 80-MHz pulse and stores four 16-bit values [Fig. 4(c)]. The module receives five inputs simultaneously: the laser-clock TTL and the photomultiplier waveform at 3.2  GS/s, two horizontal sync signals from the resonant scanners at 200 MHz, and a vertical sync signal at 200 MHz that marks each frame start. It outputs only the timing of the horizontal syncs relative to the vertical sync and the four photomultiplier values recorded at fixed delays after each pulse. With these data, four images can be reconstructed. Scanner control and image formation remain in separate software, which makes integration with any commercial microscope straightforward.

Full details of the subVIs appear in the Supplementary Material. Here, we describe how partial duplication and compression prevent data loss. Each laser pulse trigger arrives once every 12.5 ns. Assuming that the laser pulses are multiplexed by a factor of 4, the pulses that actually reach the objective lens arrive once every 3.125 ns. If the range of the fluorescence lifetime is ∼1 to 5 ns, by adjusting the cable delays, we can align the fluorescence from the first pulse so that it arrives 1 to 5 ns after the trigger. Under this condition, the fluorescence corresponding to the last pulse arrives 3.125×3+1 to 3.125×3+5  ns=12.375 to 14.375 ns after the trigger. If the system were to sample every 12.5 ns, the signal from this last pulse would be recorded after the next laser trigger. Because the FPGA cannot easily carry information over to the next trigger, we configured the system so that one measurement loop handles 15 ns of data, which corresponds to 45 samples. We then duplicated this loop and assigned one copy to even triggers [green triggers in Fig. 4(d)] and the other to odd triggers [orange triggers in Fig. 4(d)]. This parallel structure allows the FPGA to process all required data without needing to carry information over to the next trigger. This redundant sampling allowed us to provide the FPGA with sufficient flexibility for summation operations [Fig. 4(e)].

After integration the FPGA outputs four 16-bit numbers for every 80-MHz pulse. This creates a data stream of 640  MB/s. An optional accumulation count allows up to four successive pulses to be summed before transfer [Fig. 4(b)]. The per-channel rate then becomes 20 MHz, comparable to a conventional two-photon microscope. Diesel2p contains two autonomous resonant scanners that cannot be hardware-synchronized, so separate software associates the condensed photomultiplier data with the two scanner triggers in real time. Any user can add the FPGA module and its host PC to an existing microscope and obtain multiplexed acquisition. Five representative operating modes are shown in Fig. 4(f).

### 3.6 Modular Hardware and Software

To keep the system compatible with many microscopes, we adopted a modular design. The hardware accepts only synchronization and photomultiplier signals. The software performs real-time accumulation on the FPGA, sends the reduced data via Thunderbolt, and saves it to disk. Scanner control stays in the existing microscope software, so we could switch between the original controller and the high-speed sampler simply by reconnecting BNC cables. Each subVI carries out a single task, so pulse rate, acquisition speed, or spectral multiplexing can be adjusted by replacing one module. Adding this unit to a running two-photon microscope immediately enables laser multiplexing and lifetime imaging.

### 3.7 Temporal Separation of GCaMP Signals from Distinct Pathways

**Fig. 5:**
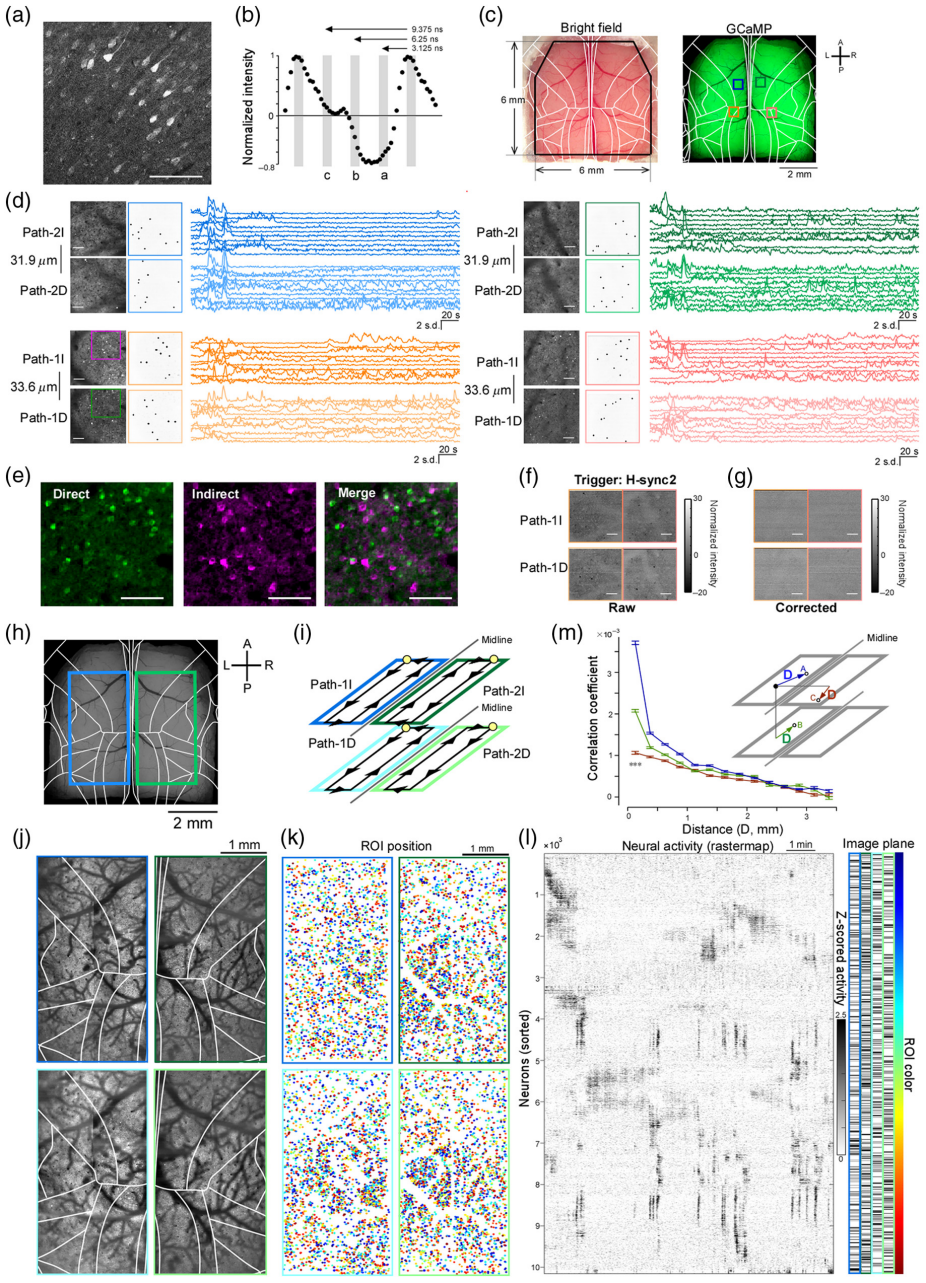
Four pathways of two-photon calcium imaging. (a) Two-photon image of a cortical slice containing GCaMP-expressing pyramidal neurons. Scale bar, 100  μm. (b) Normalized fluorescence as a function of time bins within the 12.5-ns laser period. Baseline was taken from a background region without tissue, and traces were scaled by the maximum amplitude. Three nonoverlapping windows (a)–(c), spaced by 3.125 ns, were used to estimate and correct inter-channel leakage. (c) Dorsal view of mouse cortex with a 6  mm×6  mm cranial window (left) and GCaMP epifluorescence (right). Colored squares mark the four fields used for simultaneous four-area/two-depth imaging shown in panel (d). (d) Example simultaneous calcium imaging from four areas at two depths. Signals were corrected for inter-channel leakage using coefficients derived from panel (b). Ten ROIs were randomly chosen per field, and their fluorescence time courses are shown; colors match panel (c). Each field of view (FOV) was 0.5  mm×0.5  mm, acquired at 15 Hz for 5 min. The direct pathways imaged the deeper plane of each pair. Laser power: path 1D, 115 mW, path 1I, 105 mW, path 2D, 122 mW, path 2I, 110 mW. Imaged at 15  frame/s. (e) Direct (green) and indirect (magenta) channels, separated by Δt=6.25  ns, show negligible cross-talk (merge at right). (f) To assess leakage between adjacent bins (Δt=3.125  ns), path 1 raw signals were reconstructed using the H-sync-2 trigger; the mean of 100 frames is shown. Bright vessels and dark somata reflect sign-reversed leakage. (g) Same analysis after leakage correction; vessel/soma leakage is no longer visible. (h) Bilateral two-layer imaging patches overlaid on the atlas. Scale bar, 2 mm. (i) Schematic of the four pathways (path 1D, path 1I, path 2D, path 2I). Yellow circles indicate scan start points; black lines/arrows indicate the galvanometric slow-scan trajectory. The resonant scanner sweeps orthogonal to these lines (not drawn). Each pathway images a distinct layer in one hemisphere. (j) Mean two-photon images for the two depths in both hemispheres (top and bottom panels are different depths of the same areas). Box colors correspond to pathways. Laser power: path 1D, 115 mW, path 1I, 105 mW, path 2D, 122 mW, path 2I, 110 mW. Imaged at 3.6  frame/s. (k) ROIs detected in panel (j). Colors correspond to the ordering produced by the rastermap in panel (l). (l). Fluorescence time courses from ROIs in panel (k), ordered by the rastermap. The imaging plane of each ROI is indicated in the right panel, and the lateral positions of the ROIs are shown by a color bar. (m). Pairwise correlation coefficients plotted against the lateral distance between ROIs. Blue, pairs within the same FOV; green, pairs across the two depths in the same hemisphere (z-separation of 32  μm ignored so distance is lateral only); red, inter-hemispheric pairs computed after reflecting one ROI across the midline. Error bars denote s.e.m. The closest distance bin (<0.25  mm) showed significant differences among groups (Wilcoxon rank-sum test, p<0.001) (Video 1, avi, 8.32 MB [URL: https://doi.org/10.1117/1.NPh.13.1.015013.s1]).

To image GCaMP fluorescence on four channels simultaneously, we first quantified inter-channel leakage using acute cortical slices [Figs. 5(a) and 5(b)]. With only the path 1D channel active, the mean GCaMP waveform clearly spilled into the other channels. In the *in vivo* configuration, the four pathways are sampled 3.125 ns apart (10 digitizer bins). Therefore, when GCaMP peaks on path 1D, its leakage appears sequentially on path 2D, path 1I, and path 2I at +3.125, +6.250, and +9.375  ns, respectively; by symmetry, the same ordering holds for any source channel (e.g., path 2D leaks to path 1I, path 2I, then path 1D). Let the true images be I=[I1,I2,I3,I4]⊤ and the measured images J=[J1,J2,J3,J4]⊤. Then

J=WI,

where W is a circulant matrix determined by the leakage coefficients a,b,c defined in Fig. 5(b). The demixed estimate of the true signals is obtained by

I^=W−1J

(see Sec. 2 for details).

### 3.8 Simultaneous Four-Area, Two-Plane Calcium Imaging *In Vivo*

**Fig. 6:**
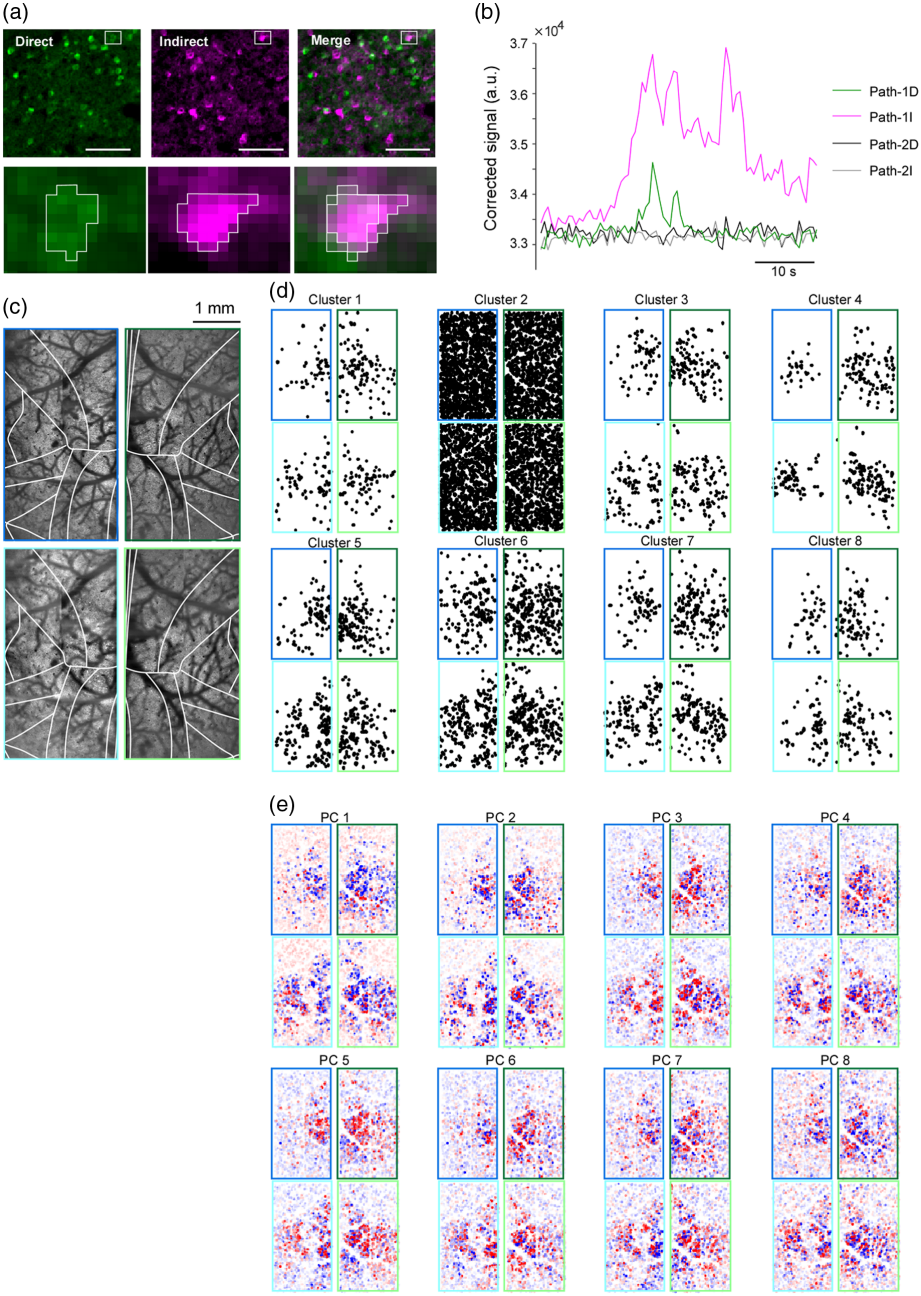
Spatial localization of neuron activity and signal crosstalk. (a) ROIs of neurons that overlapped between the path1 D and the path1 I. (b) Fluorescence changes of the ROIs specified in panel (a). The corresponding brightness values at the same spatial locations in path2 D and the path2 I are also shown. (c) Mean two-photon images for the two depths in both hemispheres [Fig. 5(j)]. (d) Results of K-means clustering of neuronal activity (K=8). Symmetrical patterns were observed between hemispheres as well as similar patterns across depths. (e) Top eight principal components from PCA of neuronal activity, shown as color plots. As with the K-means clustering, symmetric patterns across hemispheres and similar patterns across depths were observed.

To test the demultiplexing *in vivo*, we used double-transgenic mice expressing GCaMP6s broadly in cortical excitatory neurons (Ai162 × Slc17a7-IRES2-Cre). Mice were head-fixed under a 6  mm×6  mm cranial window; dorsal epifluorescence confirmed widespread expression [Fig. 5(c)]. We targeted four 0.5  mm×0.5  mm fields across the two hemispheres. Each pathway of the path1/2 imaged two of the fields, with the direct plane positioned deeper than the indirect plane by ≈32  μm. Thus, eight images (four areas × two depths) were acquired simultaneously at 15 Hz for 5 min [Fig. 5(d); Video 1]. Ten neurons per field were randomly selected to display fluorescence time courses. Visual inspection of magnified mean images revealed negligible cross-talk between the direct and indirect channels separated by 6.25 ns [Fig. 5(e)]. Moreover, the overlapping ROIs displayed clear and well-defined calcium transients without any detectable signs of leakage, further supporting the effectiveness of our demultiplexing procedure [Figs. 6(a) and 6(b)].

Because adjacent channels are separated by only 3.125 ns, we also examined leakage between neighboring bins. Although the two resonant scanners in our system have identical specifications (CRS 8 kHz), they do not synchronize, resulting in a random phase offset. In the uncorrected data, when the path 2 signals are imaged using the trigger from the path 1 resonant scanner (H-sync-1), the path 2 images are not visible. Instead, leakage from path 1 appears as a negative image due to the filter characteristics, producing an inverted contrast pattern [Fig. 5(f)]. This negative image should disappear if the crosstalk is properly corrected. Indeed, after applying the leakage correction ($W^{-1}$), the negative image was no longer visible, indicating that the correction worked as intended for adjacent channels [Fig. 5(g)]. The same result was obtained when imaging path 1 using the trigger from the path 2 resonant scanner (H-sync-2).

### 3.9 Bilateral Large Field-of-View Two-Layer Imaging

We next expanded the FOV to cover large portions of the dorsal cortex bilaterally in awake mice [Fig. 5(h)]. During each slow-scan cycle of the galvanometers, each pathway scanned one of two depths in one hemisphere [Fig. 5(i)]. Representative mean two-photon images [Fig. 5(j)], corresponding ROIs [Fig. 5(k)], and their fluorescence time series aligned by rastermap^42^ [Fig. 5(l)] are shown. Pairwise correlations of neural activity decreased with lateral distance [Fig. 5(m)]. Within-FOV, same-layer pairs showed the highest correlations, followed by within-FOV, different-layer pairs (z-offset ≈32  μm, distance computed laterally), and then interhemispheric pairs computed after reflecting one ROI across the midline. The closest distance bin (<0.25  mm) showed significant differences between groups (Wilcoxon rank-sum test, p<0.001). Note that because correlations decrease with increasing lateral distance, pairs located at different axial depths but close in lateral distance exhibit higher correlations than pairs within the same imaging plane that are separated by a larger lateral distance. These results are consistent with columnar organization. To further evaluate the simultaneously recorded neural activity, we examined how neurons with similar activity patterns were spatially distributed by applying K-means clustering and PCA. We found that neurons exhibiting similar activity tended to be located in close spatial proximity, appeared symmetrically across the two hemispheres, and showed comparable distributions across cortical depths. Together, these analyses indicate that the neural activity we recorded is reasonably consistent with expected physiological properties.^43^^–^^45^

### 3.10 Bilateral Four-Layer Imaging

**Fig. 7:**
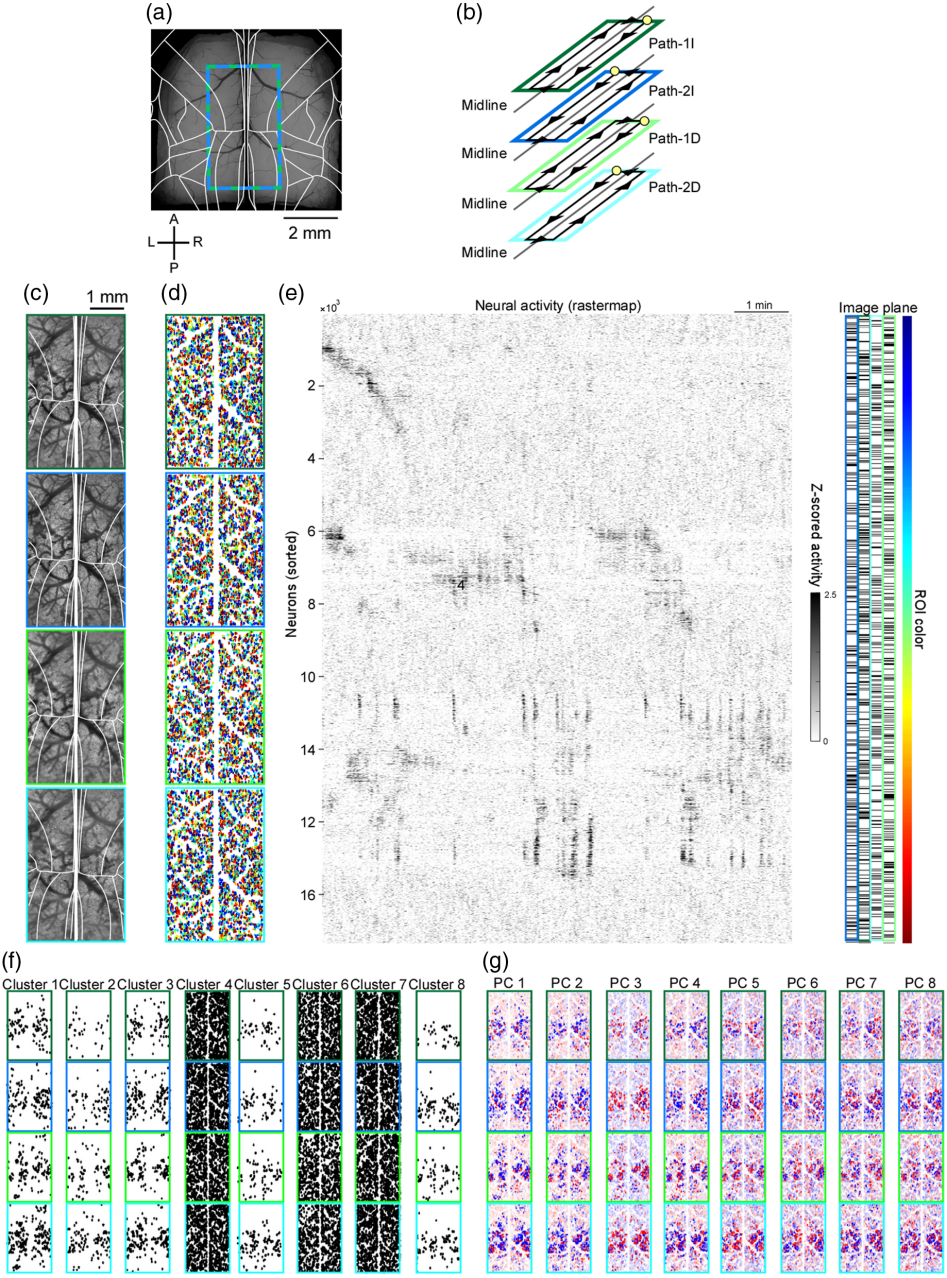
Bilateral large field-of-view four-layer two-photon imaging. (a) Imaging footprint overlaid on dorsal vasculature and the Allen Mouse Brain Atlas. Axes as indicated. Scale bar, 2 mm. (b) Acquisition schematic. The four pathways (path 1D, path 1I, path 2D, path 2I) each scan a distinct cortical layer across hemispheres. Yellow circles mark scan start-points; black lines/arrows indicate the galvanometric slow-scan trajectory. The resonant scanner moves orthogonally (not drawn). Together, the pathways provide bilateral coverage at two depths per hemisphere (four layers total). (c) Mean two-photon images from the same lateral position at four depths [corresponding to panels (d) and (e)]. Frame colors map to the pathway identity. Laser power: path 1D, 115 mW, path 1I, 105 mW, path 2D, 122 mW, path 2I, 110 mW. Imaged at 3.6  frame/s. (d) ROIs detected from the fields in panel (c). Colors correspond to the ordering produced by the rastermap in panel (e). (e) Fluorescence time courses for the ROIs in (d) (one row per ROI), ordered by the rastermap. The imaging plane of each ROI is indicated in the right panel, and the lateral positions of the ROIs are shown by a color bar. (f). Results of K-means clustering of neuronal activity (K=8). Symmetrical patterns were observed between hemispheres as well as similar patterns across depths. (g) Top eight principal components from PCA of neuronal activity, shown as color plots.

Finally, to image four distinct depths simultaneously, we introduced a focal offset between path 1 and path 2 by translating the paired achromatic doublet placed immediately upstream of the path 2 Galvo-X2. This lens pair is marked with an asterisk in Fig. 1(a). This adjustment of the lens position caused a focal shift of ∼15 to 20  μm, but it did not noticeably degrade the optical performance. In practice, this shift is much smaller than the focal movement introduced by the ETL, and simulations also confirmed that the optical performance was largely preserved. Combined with depth tuning of the indirect paths via the ETL, this yielded four independent focal planes [Figs. 7(a) and 7(b)]. Using this configuration, we recorded four layers across the medial regions of both hemispheres simultaneously [Figs. 7(c)–7(e)]. As in the two-layer imaging, K-means clustering and PCA revealed that neurons with similar activity patterns tended to be located in close spatial proximity [Figs. 7(f) and 7(g)].

### 3.11 Large FOV 2pFLIM

Because our digitizer operates at high speed, the same system can be used not only for multiplexing but also for fluorescence lifetime imaging. Therefore, we performed two-photon fluorescence lifetime imaging using the same high-speed digitizer [Fig. 3(f)]. Sections of Convallaria, with endogenous fluorescence lifetime mirrors tissue architecture, are widely used as FLIM standards. In our setup, the 12.5 ns interval between successive 80 MHz laser pulses was divided into 40 temporal bins. Consequently, we first acquired a stack of 40 images with the same FOV, each averaged over 1000 frames. Comparing pixel intensities across these 40 frames yielded fluorescence signals with a temporal resolution of 12.5  ns/40. Because the system can store data from only four bins per acquisition, imaging had to be repeated ten times to obtain the full 40-bin dataset.

**Fig. 8:**
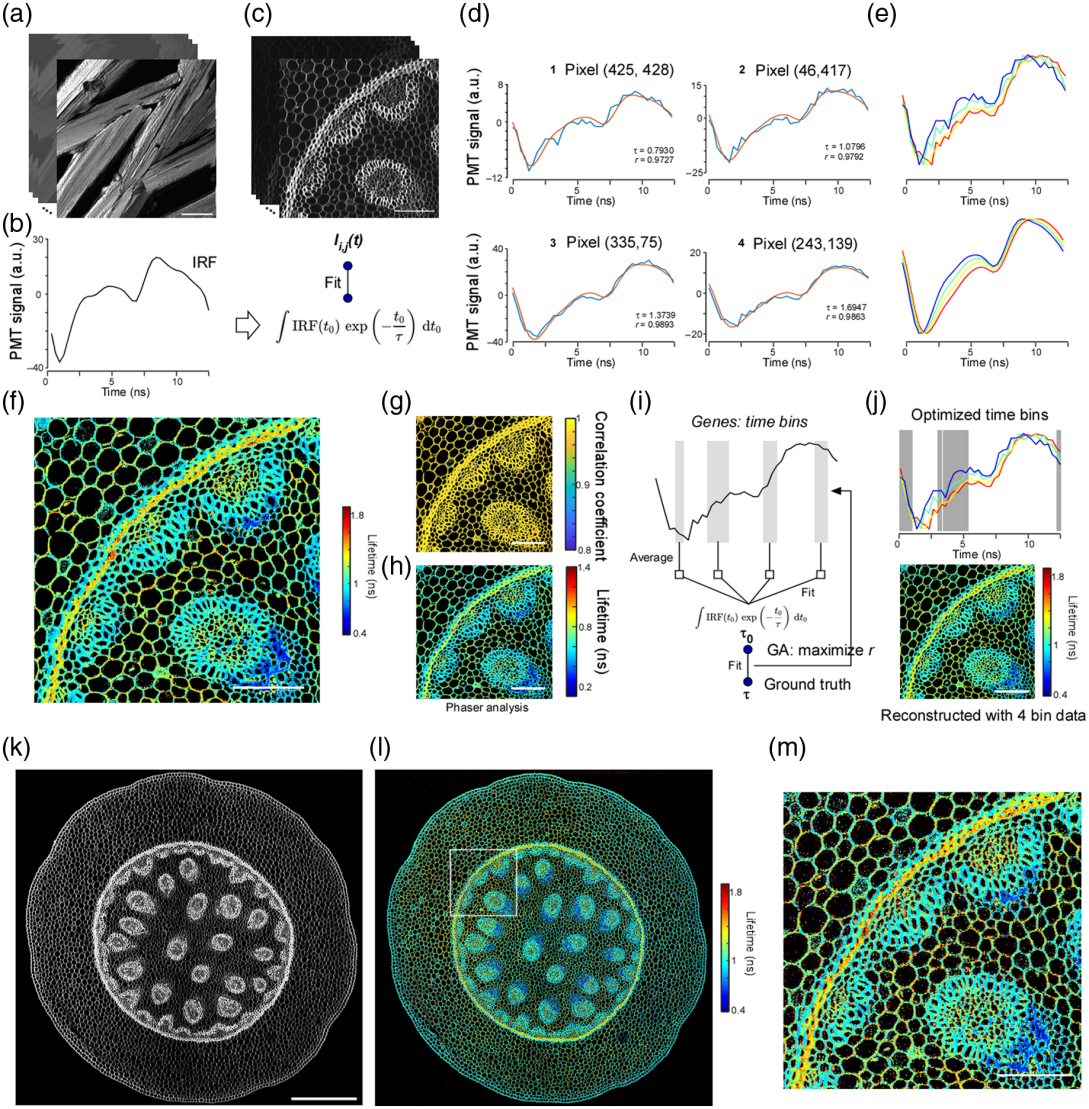
Large FOV two-photon lifetime imaging with Diesel2p. (a) Two-photon imaging of urea crystals. The signal is second-harmonic generation and therefore represents the system impulse response function (IRF). Forty consecutive images visualize the 12.5 ns pulse period. Scale bar, 100  μm. (b) Average of the forty images in panel (a), giving the IRF. (c) Two-photon fluorescence imaging of Convallaria, top part. Forty consecutive images visualize the 12.5 ns pulse period. In the bottom part, each pixel intensity trace is fitted with the convolution of the IRF in panel (b) and a single-exponential decay, yielding the fluorescence lifetime τ. (d). Temporal traces and fits for four representative pixels taken from panel (c). (e) Overlay of the four traces and fits shown in panel (d), colored according to the lifetime scale in panel (f). (f) Lifetime map in which pixels brighter than the background are colored by their fitted τ value. Scale bar, 100  μm. Two hundred images were used to compute the fluorescence lifetime. The same number of images was used for panels (h), (j), (m), and (l). (g) Pixel-wise correlation coefficient of each fit in panel (f). Most pixels show a value greater than 0.95. Scale bar, 100  μm. (h) Lifetime image obtained by phasor analysis of the same data set. Scale bar, 100  μm. (i). Procedure for selecting four time bins that allow lifetime estimation from only four measurements. A genetic algorithm optimized the bin positions using pixels in the lower-left triangle of the image. (j) Lifetime image produced with the four optimized bins from panel (i). High accuracy was obtained even in the upper-right triangle, which the algorithm did not use for training. Scale bar, 100  μm. (k) Large FOV two-photon image of the entire Convallaria section. Scale bar, 500  μm. (l) Lifetime map corresponding to panel (k). (m) Magnified view of the boxed region in l; this region corresponds to panels (f), (h), and (j). High estimation accuracy was retained across the large field of view. Scale bar, 100  μm.

We then characterized the system response using the second-harmonic generation (SHG) signal from urea crystals [Figs. 8(a) and 8(b)]. As SHG is generated with zero temporal lag, it provides the instrument response function (IRF). Convolving this IRF with a single-exponential decay of lifetime τ and fitting it to the Convallaria data allowed us to determine the fluorescence lifetime of each pixel [Figs. 8(c)–8(e)]. The Convallaria signals were accurately described by the IRF-convolution model, enabling resolution of subtle lifetime differences. Applying this fitting to all pixels produced a fluorescence-lifetime image [Fig. 8(f)]; most pixels exhibited a correlation coefficient >0.95 between the model and the data [Fig. 8(g)]. Phasor-plot analysis yielded an equivalent lifetime map, aside from a slight phase shift [Fig. 8(h)]. Together, these results confirm that our FPGA-based system is well suited for lifetime imaging.

To obtain a lifetime image in a single scan, we optimized the choice of four time-bins available per excitation pulse. A genetic algorithm was used to identify the four-bin combination that best reproduced the lifetimes determined from the complete 40-bin dataset [Fig. 8(i)]. The selected bins generated lifetime images virtually indistinguishable from the 40-bin reference [Fig. 8(j)].

**Fig. 9:**
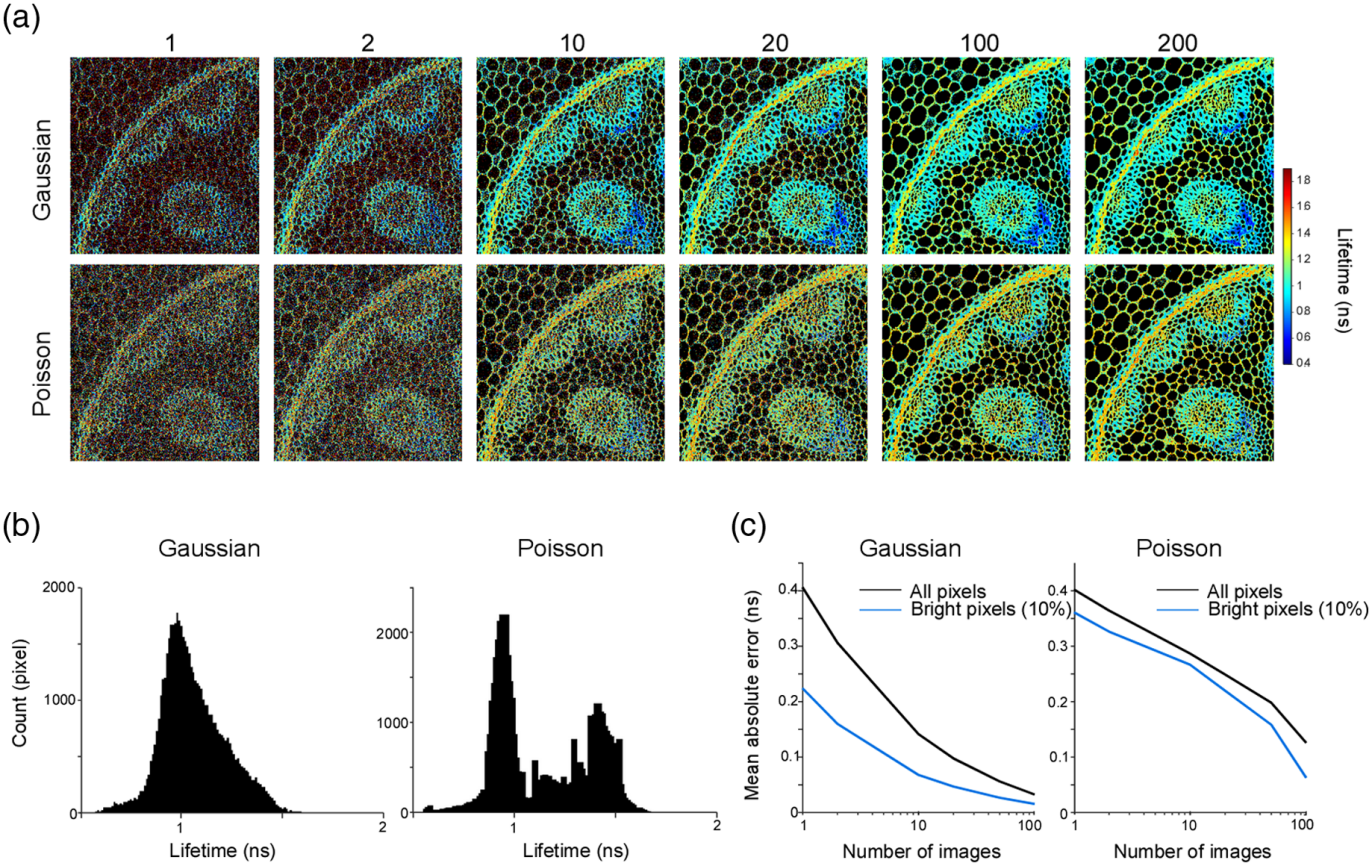
Relationship between fluorescence lifetime and the number of images used for its estimation. (a) Lifetime images simulated under assumptions of Gaussian noise or Poisson noise are shown in the order of the number of images used for the estimation (numbers above each panel). (b) Histograms of the estimated lifetimes under Gaussian and Poisson noise assumptions. The histograms were computed using lifetime values estimated from 200 images. (c) Assuming the lifetime image computed from 200 images represents the ground truth, the mean absolute error (MAE) was plotted as a function of the number of images used for lifetime estimation. The MAE was computed using either all pixels (black line) or the top 10% brightest pixels (blue line) under both Gaussian and Poisson noise assumptions.

To acquire a lifetime timelapse, it is necessary to determine the number of images required for accurate lifetime estimation. We examined the estimation error as a function of the number of images used to compute the lifetime [Figs. 9(a)–9(c)]. Because photon counts are low in many pixels, a Poisson noise model is more appropriate than a Gaussian model. We therefore also evaluated a method that maximizes the Poisson likelihood (see Sec. 2) and compared the results. We found that lifetime estimates continued to improve when several hundred images were averaged. However, high-intensity pixels produced an absolute error of only ∼0.2  ns. Maximizing the Poisson likelihood did not improve the estimation accuracy, which suggests that factors other than photon-count noise, such as additional measurement noise, may dominate the error. These findings support the feasibility of visualizing lifetime dynamics using wide-field two-photon microscopy. However, several factors require careful consideration, including the fluorescent sensor used, the laser power, the size of the imaging field, and the pixel dwell time.

Finally, the FOV was enlarged to encompass the entire Convallaria section, and images of comparable quality were obtained [Figs. 8(k)–8(m)]. These findings demonstrate that integrating fluorescence lifetime imaging with the Diesel2p mesoscale microscope enables simultaneous acquisition of wide-field lifetime data.

## 4 Discussion

In this study, we split the 80 MHz laser beam into four paths, enabling four-field mesoscale imaging with the widely used 80 MHz light source. We achieved efficient, loss-free signal processing by subdividing the 12.5 ns inter-pulse interval into 40 bins (0.31 ns each) and compressing the data on the fly with an FPGA. Using this system, we were able to perform large-scale calcium imaging *in vivo* of the bilateral wide dorsal cortex across up to four planes. We also demonstrated lifetime imaging, confirming sub-nanosecond resolution using Convallaria sections with a large FOV.

Although the Diesel2p is the only mesoscale microscope with an open-source optical path, we have additionally released fully open-source control software and acquisition hardware. Our new FPGA system has a modular architecture that is independent of the scanner-control electronics, so it can be plugged into any existing two-photon microscope via a BNC cable and used immediately. Multiplexing and lifetime imaging are therefore available not only on Diesel2p but also on conventional systems, and compatibility with the ubiquitous 80 MHz laser lowers the adoption barrier. Excitation efficiency on Diesel2p is close to its power limit with four fields, but further multiplexing should be possible with a lower-repetition-rate laser or adaptive optics. Although we do not achieve the temporal resolution of TCSPC, the ability to record multiple FOVs or large FOVs simultaneously offers a significant increase in acquisition speed. The FPGA code is clearly modular and written in the standard National Instruments LabVIEW software, which facilitates extensions such as machine-learning-based image analysis,^46^^,^^47^ integration with optical stimulation, and closed-loop or BMI experiments.^48^^,^^49^

The system developed by Jerry Chen’s group^4^ used two laser colors; our design also supports dual-color excitation, making palette expansion straightforward. Techniques such as reverberation and light-beads can deliver even higher multiplexing, but when Diesel2p is driven by an 80 MHz laser, four beams are the practical limit, making our system a realistic best solution. Combining Diesel2p with a reverberation circuit, a light-beads module,^21^^,^^22^ or a FACED module^20^ is nevertheless possible. For instance, lowering the repetition rate to ≈10  MHz would theoretically allow an eightfold increase, yielding 32 beams—likely close to the *in vivo* limit for two-photon excitation. Multipixel detectors such as SiPM arrays can mitigate crosstalk and raise the ceiling further, yet laser-induced brain damage will ultimately constrain *in vivo* power.

With FLIM now feasible on a large FOV two-photon microscope, a diverse set of FRET sensors, including cAMP,^50^ PKA,^51^ CREB,^52^ CaMKII,^53^ Ras-GTPase,^26^^,^^28^ ERK/MAPK,^54^ and PI3K/PTEN^55^ can be applied to large-scale *in vivo* brain imaging. Because FLIM is robust against biological motion, it is valuable not only for calcium indicators with pronounced lifetime changes^56^ but potentially also for membrane-voltage sensors.^57^ One can conduct macroscale RCaMP imaging on optical path 1 while simultaneously performing spine-level cAMP or Ras FLIM imaging on Path-2, bridging macroscale activity with local biochemistry across multiple time scales. The combination of large FOV coverage and lifetime imaging should accelerate multisignal detection in cleared brains, speeding transcriptomic, and connectomic mapping exponentially. Label-free measurements of NAD(P)H and FAD^58^^,^^59^ and separation of SHG signals^60^ are also possible. Beyond the brain, *in vivo* FLIM has been demonstrated in retina,^61^ lung,^62^ intestine,^63^ pancreas,^64^ liver, and kidney,^65^ with rapid progress toward human applications. In disease research, the platform enables studies of cancer,^66^ Alzheimer’s disease,^67^ diabetes,^68^ the immune system,^69^ and vascular disorders. Thus, broad adoption of our open-source system promises wide-ranging contributions from basic science to clinical medicine.

Finally, we describe the limitations of our system. Multiplexing an 80 MHz laser into four beams requires fluorescence detection within a 3.125 ns time window. Although channel crosstalk could be corrected when photon counts were sufficiently high, this was likely due to several favorable conditions. First, we used relatively high laser power, ∼100  mW, which could yield sufficient fluorescent photons per pulse. Second, the spatial distribution of the neurons we imaged was relatively sparse, leading to only a small number of overlapping signals. Third, in cases where overlapping activity occurred, those neurons may not have been detected as ROIs by Suite2p,^41^ which assumes local correlated pixels within a single neuron. However, none of these conditions fundamentally eliminates crosstalk. Therefore, for our approach, it is necessary to evaluate, for each sample, whether crosstalk correction is feasible by examining whether the intensity of ROIs is correlated across overlapping channels. Because 80 MHz lasers are the most widely used, we focused on achieving four-way multiplexing under this constraint. Ideally, however, an approach that increases the temporal separation between pulses would be more appropriate.^4^^,^^22^^,^^43^

## 5 Appendix: Supplementary Material

Supplementary Video 1 shows data from Figs. 5(c)–5(g). Images of direct (magenta) and indirect (green) pathways were superimposed, and those of four FOVs were combined. Total video length: 54 s. Supplementary Material includes supplementary discussion, FPGA operation mechanisms, instruction of the LabVIEW software, and modified optical parameters for Diesel2p.

## Supplementary Material

10.1117/1.NPh.13.1.015013.s01

10.1117/1.NPh.13.1.015013.s1

## Data Availability

The LabVIEW script is available at https://www.dropbox.com/scl/fo/kh5q80fw86ca81k0f0yy5/ADaUPb8EX2jGyErOhmySVrk?rlkey=mgahmvfkz9wltbxcnuwsr1njs&dl=0. Further development of this software may be supported by one of the authors, Osamu Fujioka (Trionix Inc.). If you have any questions, please do not hesitate to contact the corresponding author. The updated optical parameters of the Diesel2p system are provided in the Supplementary Material. Additional modifications may be supported by another author, Satoshi Suitoh (KYOCERA SOC), so please feel free to contact the corresponding author regarding these matters. Requests concerning the lifetime estimation algorithm or related analyses may also be directed to the corresponding author. The experimental datasets are publicly available on Figshare: https://doi.org/10.6084/m9.figshare.31449091.
